# Growing pains with kratom: experiences discussed in subreddits contrast with satisfaction expressed in surveys

**DOI:** 10.3389/fphar.2024.1412397

**Published:** 2024-06-14

**Authors:** Jeffrey M. Rogers, Kayla Colvin, David H. Epstein, Oliver Grundmann, Christopher R. McCurdy, Kirsten E. Smith

**Affiliations:** ^1^ San Diego State University/University of California San Diego Joint Doctoral Program in Clinical Psychology, San Diego, CA, United States; ^2^ Real-world Assessment, Prediction, and Treatment Unit, National Institute on Drug Abuse Intramural Research Program, Baltimore, MD, United States; ^3^ College of Pharmacy, Department of Medicinal Chemistry, University of Florida, Gainesville, FL, United States; ^4^ Department of Psychiatry and Behavioral Sciences, Johns Hopkins University, Baltimore, MD, United States

**Keywords:** kratom, kratom dependence, kratom extracts, emerging drugs, harm-reduction

## Abstract

**Background:**

“Kratom” refers to an array of bioactive products derived from *Mitragyna speciosa*, a tree indigenous to Southeast Asia. Most kratom consumers report analgesic and stimulatory effects, and common reasons for use are to address mental and physical health needs, manage pain, and to reduce use of other substances. Natural-history studies and survey studies suggest that many kratom consumers perceive benefits from those uses, but such studies are unlikely to capture the full range of kratom-use experiences.

**Methods:**

We collected text data from Reddit posts from 2020-2022 to qualitatively examine conceptualizations, motivations, effects, and consequences associated with kratom use among people posting to social media. Reddit posts mentioning kratom were studied using template thematic analysis, which included collecting descriptions of kratom product types and use practices. Network analyses of coded themes was performed to examine independent relationships among themes, and between themes and product types.

**Results:**

Codes were applied to 329 of the 370 posts that comprised the final sample; 134 posts contained kratom product descriptions. As Reddit accounts were functionally anonymous, demographic estimates were untenable. Themes included kratom physical dependence (tolerance, withdrawal, or use to avoid withdrawal), perceived addiction (net detrimental effects on functioning), and quitting. Extract products were positively associated with reports of perceived addiction, dependence, and experiences of quitting kratom. Many used kratom for energy and self-treatment of pain, fatigue, and problems associated with opioid and alcohol; they perceived these uses as effective. Consumers expressed frustrations about product inconsistencies and lack of product information.

**Conclusion:**

As in previous studies, kratom was deemed helpful for some and a hindrance to others, but we also found evidence of notable negative experiences with kratom products that have not been well documented in surveys. Daily kratom use may produce mild-moderate physical dependence, with greater severity being possibly more common with concentrated extracts; however, there are currently no human laboratory studies of concentrated kratom extracts. Such studies, and detailed kratom product information, are needed to help inform consumer decision-making.

## 1 Introduction

Kratom (*Mitragyna speciosa*) and kratom-derived products are proliferating internationally, and in the United States (US; Grundmann et al., 2023). Current pharmacological evidence suggests that kratom can exert opioid-like and stimulant-like effects, with preliminary evidence for opioidergic, adrenergic, and serotonergic receptor actions (McCurdy et al., 2024). These products are not currently regulated by the US Food and Drug Administration (U.S. Food and Drug Administration, 2024) and are the focus of intense debate. Surveys of consumers in the US suggest that kratom use can increase perceived quality of life, either by seeming to alleviate specific physical or psychological problems or by seeming to increase energy and productivity (Grundmann, 2017; Smith et al., 2021a; Smith et al., 2022b; Smith et al., 2022d). While kratom has only recently proliferated in the US, rural populations in Southeast Asia have historically used kratom as a remedy for common ailments, to ameliorate fatigue, and as an adjunct to social and religious events (Singh et al., 2017). Reports of perceived benefits have not been tested in randomized trials but have been recently systematically documented, along with other effects, using intensive longitudinal assessment in consumers’ daily lives (Smith et al., 2024a) and laboratory-based direct-observation of the acute effects of consumers’ self-selected dose (Smith et al., 2024b).

Yet, even these data contain indications of kratom-associated problems: many or most daily users report some symptoms of physical dependence, and some appear to meet criteria for addiction—that is, kratom-use disorder, by the criteria in the *Diagnostic and Statistical Manual, Fifth Revision* (DSM-5) (American Psychiatric Association, 2013).^1^ Evidence regarding clinical adverse effects from use of kratom and kratom-derived products is primarily documented in case reports, and toxicities appear mostly in the context of polydrug exposures (Eastlack et al., 2020; Grundmann et al., 2023). In systematic reviews, we have found shortcomings and ambiguities in case reports of kratom-related adverse events (Smith et al., 2022a) and kratom addiction (Feldman et al., 2023). Shortcomings notwithstanding, it is impossible to ignore accumulating reports for both the perceived benefits of kratom products and for their seeming capacity to harm.

That is the context that leads us to present the following report, which we originally planned to publish in 2023. We had initially framed it as an update on a rapidly evolving situation of kratom product diversification and proliferation, accompanying the diversification of self-reported experiences, but we now offer this report for a more enduring purpose: to help ensure that peer-reviewed publications about kratom do not reflect the polarization seen in popular discourse about it. If specific groups of investigators consistently emphasize only positive or only negative aspects of kratom use, the credibility of their contributions may suffer. Our recent publications about kratom (Smith et al., 2024a) largely illustrated how kratom use appears to benefit many consumers. Most of our participants had found products and dosage patterns that appeared, during the timeframe assessed, satisfactory to them and not detrimental to their health or daily functioning. We stand by those findings, but we have always noted that they are not the whole story of kratom products. Here, we give voice to consumers who believe that their relationship with kratom was not unambiguously positive or beneficial, and to some who believed kratom caused them harm.

The social-media content analyzed here is also a document of the last years in which Reddit data were publicly available for collection and analysis. On 18 April 2023, Reddit began to restrict access to their API as part of an initial public offering, and our data-collection method is no longer feasible for future studies of Reddit posts. These data on kratom capture a shift in perceptions—a growing awareness among a minority of consumers that product information was insufficient and that kratom use could lead to problems.

We aimed to examine the product types reported in kratom-related Reddit posts and associations between product type and other themes that emerged from text segments in the posts. Within text segments, we qualitatively examined 1) conceptualizations of kratom, 2) self-reported motivations for kratom use and how effective people judged kratom to be for these motivations, 3) self-reported subjective effects (both positive and negative) of kratom and whether other reported factors were associated with these, 4) descriptions of kratom consumption patterns, including use of kratom with other substances, 5) descriptions of kratom-use disorder and/or other consequences associated with use, and 6) emergent themes related to kratom use (see method 2.2 *Codebook Generation and Text Analysis*).

## 2 Methods

### 2.1 Data collection

We collected individual post text and metadata from Reddit, a western-centric social-media platform comprising discrete content boards called “subreddits,” which host topic-relevant news, media content, and discussion threads. Post text data from Reddit discussion boards, called “threads” provide rich descriptions of personal experiences, and unlike other platforms (e.g., Twitter, Facebook) at the time of data collection, Reddit’s data were publicly accessible via their application programming interface (API). Data from discussion threads were collected in real time using the R package RedditExtractoR (Rivera, 2015), which functioned like a wrapper for accessing Reddit’s API. We identified kratom-related discussion thread URLs by pooling data from iterative calls to Reddit’s built-in search function, using a comprehensive list of kratom terms and common misspellings observed in our previous Reddit analysis. With the R package quatenda (Benoit et al., 2018), text from each discussion thread was processed into corresponding lists of unique word tokens, which we used to exclude posts containing no word tokens from our initial search list. The final sample contained 370 Reddit threads from July 2020 to January 2022. For additional methodological description, including discussions of reddit’s nuances, see (Smith et al., 2021b).

Procedures were performed with respect to applicable laws and reflect the practices we have used in our prior investigations into attitudes toward kratom (Smith et al., 2021a), tianeptine (Smith et al., 2021b), and kava (Pont-Fernandez et al., 2022). Because this study used only data that were publicly available, it was deemed exempt from full review by the National Institutes of Health Institutional Review Board. The public, largely anonymous nature of post data meant that there was no method for obtaining consent, even if consent had been mandated. A central, defining feature of Reddit, compared with other social media platforms, is its culture of anonymity (Van Der Nagel and Frith, 2015; Robards et al., 2021). Platform-suggested usernames are random and user profiles omit legal names, ages, countries of origin, and association lists. Additionally, Reddit also maintains policies against the posting of user’s private information in an attempt to identify other users, also referred to as “doxing” (Copland, 2021). Despite Reddit’s provision of relative anonymity, researchers have raised ethical concerns about publishing usernames and direct quotes from the platform, as this information may be connected with other post information to potentially identify individual Reddit users (Gliniecka, 2023). In selecting the degree of “disguise” for the present study, we omit username and profile information to disconnect selected quotes from individual Reddit users (Reagle, 2022). Relatedly, one of our previous papers, in which we used Reddit post data about kratom, was requested by and provided to a moderator of a kratom subreddit. This occurred during our recruitment for an unrelated kratom study (Smith et al., 2024a), for which many Reddit posters screened and enrolled. Likewise, some of the authors (CRM) have participated in Reddit “Ask Me Anything” threads. We note this engagement to emphasize the respect we have for kratom subreddit communities. We want to underscore the seriousness with which we undertake our data collection and analyses and our desire to give posters a voice in peer-reviewed literature. No quotes that appear in this manuscript contain personally identifiable information.

### 2.2 Codebook generation and text analysis

Reddit texts were examined using thematic analysis (Brooks et al., 2015), a process-based qualitative method which aims to develop a template (coded set of themes or “codebook”) from a qualitative dataset and subsequently use that template for text coding. Our approach was informed by Braun and Clark’s writings on thematic analysis (Braun and Clarke, 2022). First, authors (KS, KC, and JR) familiarized themselves with the data by independently reading it. Although we had assumptions regarding content we might encounter (e.g., kratom use for self-treating psychiatric symptoms) based on prior findings (Coe et al., 2019; Bath et al., 2020; Garcia-Romeu et al., 2020; Smith et al., 2022d), we made note of these and novel/emergent themes in the data. Throughout this phase, we met to generate initial themes and refine them into a codebook of defined themes. KS and KC then conducted another reading of the texts, applying codes to the data. Codes were independently applied using MAXQDA 2021 (VERBI Software, Berlin). Novel content or emergent themes not captured during data familiarization and initial coding were open to coding for later review; raters (KS and KC) highlighted infrequent but noteworthy text segments for further discussion (none warranted a new code). Coding discrepancies were resolved during two conferences in which codebook themes were refined into the 9 themes/13 subthemes described in Table 1. This resulted in two additional rounds of independent coding to ensure that codes were applied appropriately based on the agreed conceptualization, meaning any residual rater disagreement did not reflect confusion regarding a specific construct (e.g., tolerance). In service of our primary aim, we examined kratom product types from text segments in which people described using kratom and categorized products mentioned as either kratom extracts, kratom powder/crushed leaf, or kratom tea. During conferencing, we noted that code discrepancies occurred for topics with which raters had differing familiarity (e.g., chemistry, dependence symptoms: KS has a clinical background; KC, a preclinical background). Though interrater agreement and reliability measures are not always indicated for thematic analysis, we report interrater agreement as percentage of total codes applied to text segments and Cohen’s kappa coefficient. Our use of line-by-line coding was not to relate our method to grounded-theory concepts or procedures; we find this method particularly helpful for facilitating codebook development and for quantifying agreement after formal coding. Text segments selected for inclusion in Table 2 and Table 3 were those that coders agreed upon as either representative of a theme broadly or of individual variability within a given theme.

**TABLE 1:** Kratom product types identified at the level of Reddit post and coded themes identified at the level of text segment, listed in descending order of total frequency (Total), applied to the sample of Reddit posts (*N* = 370) made between July 2021 to January 2022.

Kratom product types reported in reddit posts			N Posts	% total	Agree	Disagree	Total	% agree
Leaf Material (powder, crushed leaf, filled capsules)			63	17.03%	349	21	370	94.30%
Extracts (liquid extract shots, edibles, home preparations)			56	15.14%	327	43	370	88.40%
Brewed Tea			33	8.92%	346	24	370	93.50%

Coded Themes in Text Segments	Descriptions				Agree	Disagree	Total	% Agree
Dependence/Addiction	Descriptions of kratom use despite consequences, functional/role impairment, or other medical/psychological problems				**288**	**65**	**353**	**81.59**
Withdrawal	Mentions withdrawal from kratom, fear of withdrawal, plans for dealing with withdrawal, specific symptoms				136	32	168	80.95
Tolerance	Escalating dose or needing to re-dose more frequently to achieve desired effects				53	17	70	75.71
Kratom Science at Home	Research discussions, potentiation/optimization, and at-home extractions				**168**	**43**	**211**	**79.62**
Free-form scientific regurgitation	Attempts to share or discuss scientific knowledge (e.g., links to papers; scientific findings)				76	22	98	77.55
Potentiation/optimization	Altering use methods for greater perceived intensity or duration of effects				54	17	71	76.06
Alkaloid extraction methods	Processes for isolating kratom alkaloids from plant material				37	9	46	80.43
Self-treatment Motivations	Kratom use to self-treat physical and/or mental health symptoms				**183**	**22**	**205**	**89.27**
Treat SUD/SUD Symptoms	Use to treat problems arising from the use of other substances and time-frame of other substance replacement (long/short-term)				90	9	99	90.91
Long-term drug replacement					42	11	53	79.25
Short-term drug replacement					28	5	33	84.85
Self-treat Pain	Acute and chronic				56	19	75	74.67
Self-treat Psychiatric Symptoms	General or social anxiety, depressive symptoms, trauma symptoms; may be reported in vague terms like stress-related insomnia, rumination, general internalizing issues, psychological difficulties following trauma				50	6	56	89.29
Quitting/Tapering/Reducing Use	Personal experiences with quitting, plans to quit, or desire to quit kratom use				**179**	**24**	**203**	**88.18**
Negative/Unwanted Effects	Reports of negative experiences not attributable to withdrawal symptoms (e.g., puffy eyes, dry mouth, nausea, shortness of breath, constipation, abdominal pain, dizziness, drowsiness, fainting, irritability, etc.)				**106**	**21**	**127**	**83.46**
Enhancement Motivations					**92**	**20**	**112**	**82.14**
Recreation	Use to feel the effects of kratom				36	12	48	75
Nootropic	Cognitively enhancing (e.g., for alertness, focus, attention)				34	12	46	73.91
Ergogenic	Physically enhancing (e.g., for energy or physical endurance, as a pre-workout)				26	6	32	81.25
Polydrug use involving kratom	… in real-time or within a reasonably proximal period of time				**87**	**14**	**101**	**86.14**
Kratom Policy Discussion	Post mentions kratom regulation/position statements by the FDA, DEA, WHO, or state level government				**60**	**12**	**72**	**83.33**
Kratom Industry Discussion	Quality differences/differing effects (or lack thereof), Quality control (good manufacturing practices, GMP), price, availability, marketing, vendor transparency in labeling, or lab-testing				**46**	**13**	**59**	**77.97**
Total					1,209	234	1,443	83.78
**Kappa = 0.82**		**R1 (+)**	**R1 (−)**		P (observed) = Po = a/(a + b + c) = 0.84			
	**R2 (+)**	1,209^a^	99^b^	1,308	P (chance) = Pc = 1/N Themes = 0.11			
	**R2 (−)**	135^c^	0	135	Kappa = (Po - Pc)/(1 - Pc) = 0.82			
		1,344	99	1,443				

Themes were identified at the first level (in bold text) and then further specified at the second level (e.g., Dependence/Addiction as level 1, specific mentions of withdrawal and tolerance as level 2 specifiers). Reported are interrater agreements (vs disagreements), total codes applied, and agreement percentage, which were calculated for level 1 themes (in bold text).

**TABLE 2:** A sample of quotes from Reddit posts (*N* = 370) made between July 2020 to January 2022 detailing kratom dependence, addiction, quitting, extract use, and kratom potentiation and optimization methods.

Kratom dependence, addiction, and quitting	Extract use	Kratom potentiation and optimization
*“An important lesson I’m trying to teach myself, and the crux of the message of this post, is that for some of us quitting kratom isn't a miracle cure either. Quitting kratom isn't going to cure/fix whatever ailment you were experiencing before addiction. Quitting alone isn't going to get some of us to the “100%" that other users claim. Yes, quitting even for a few days is a HUGE accomplishment and you should be super proud! And sure, we may start to feel better, and things will improve but my thought is that for those of us who used K to fill a void, that void will likely still exist after we quit.”*	*“Started using kratom leaf, and over time, built a tolerance. I found extract capsules and loved it (a little too much). Worked up to 5 capsules a day over a period of 6 months. I’ve been on the same dosage for roughly 1 year. Today I found out that it’s a 50:1 extract ratio (blew my fucking mind when I heard that), meaning that theoretically each capsule (0.5 grams) is equal to 25 grams of kratom leaf. I did the math after I heard this information, which would mean I’ve been taking the equivalent of 125 grams of kratom leaf per day.”*	*“Coffee is the most common add-on stim[ulant] wise that I see added onto kratom and I wish to enlighten some of you on a better, less jittery alternative known as Yerba Mate (and it’s cousin, Guayusa). Yerba mate contains high levels of caffeine* … *but ZERO jitters, it actually even relaxes me (likely due to a combination of high levels of theobromine and theophylline in Yerba, Guayusa contains the theobromine too, but it instead of phylline it contains l-theanine) and is MUCH more euphoric than coffee.”*
*“I normally always take several days off kratom per week to make sure I am not becoming dependent or relying on the plant to heavily. That said, I have never found kratom to be particularly addictive.”*	*“Overall, the [extract] high feels like taking prescription pills or illicit opiates. Nice and pleasant euphoria with rushes of body orgasms, feels very relaxing and comfy. Better handled carefully.”*	*“I also take kratom daily and this extract potentiates the effects substantially. I purchased this extract after reading a paper about gotu kola antagonizing CCK receptors. CCK antagonism can lower tolerance to opioids, and I was curious to see if this extract could lower kratom tolerance.”*
*“Kratom came into my life and seemed to be the miracle supplement I was looking for. Such calm focus and good energy* … *That’s how I viewed it, as a supplement not a drug. First mistake! Kratom quickly led me down the addiction road. After 5 months of steadily increasing usage, I was sooo hooked. I loved what it did for me. However, the growing sexual dysfunction and ever-increasing insomnia and other side effects were taking a toll and screaming WARNING!!* … *I woke up and said: ‘no kratom today.’ Since my usage had gotten heavy including extracts, I went into withdrawal immediately.”*	*I had been taking Kratom daily for a few years and never had an issue. However, about a year ago I walked into my local smoke shop and finally decided to splurge a bit and bought the two [extract shots] for twenty. It all went downhill after that. Started out as a once a week deal then quickly turned into a $20 dollar a day habit. Here I am a year later, and I finally kicked it. I’ve been off Kratom completely for over 3 months. However, I did get some regular white Bali caps last week and it seems like the concentrates took all the joy out of regular Kratom. I’d highly recommend staying away from any of those products. Less really is more in the Kratom world.”*	*“* … *I have to take some words for precaution, because the result is more powerful than the usual kratom experience. Also, it requires chemicals that are not beneficial for your health to say the least, if handling them without any kind of protection.”*
		*“It has been 20 days since I created a mixture of kratom red vein leaf powder, white vinegar, and isopropyl alcohol; shaking daily as the mixtures separate upon resting. The white vinegar takes the mitragynine + 7-hydroxymitragynine, then upon shaking, the isopropyl alcohol takes mitragynine + 7-hydroxymitragynine from the vinegar.”*
*“I just wanted to let everyone know that kratom, if abused, is not always harmless.”*		*“After taking various powders, capsules and extracts for years, I finally switched it up and traveled down the potentiation path. All I can say is wow, I am feeling top nine right now.”*
*“I mostly wanted to put this together because right now I’m 2 weeks sober [from kratom] with 1 relapse 5 days ago and all I can think about is kratom. I am craving for hours every day but reading these posts makes my sober brain convince me I really shouldn't because I really wanna quit.”*	*“Well, I can’t digest the capsules, what about these liquid shots? Oooooh they don’t hurt my stomach. Great buzz. Well, I’m hooked. Started with one every other day. Then once a day* …. *then almost 2 years later it was FIVE SHOTS a day. Soon as I open my eyes in the morning, first thing comes to mind, shot of kratom. My whole life revolves around the 2.5-hour intervals between taking kratom.”*	*“Kanna and weed/alcohol will potentiate each other. Other good synergies include coffee, kratom, Phenibut, damiana or blue lotus.”*
		*“Fuel-efficient kratom. Ingredients for full technique: black pepper, vinegar OR lemon juice, lime juice, turmeric, cayenne pepper, grapefruit juice, Greek yogurt, Vitamin C.”*
*“I feel like me again. Kratom numbed me, but the real me was there all along. There are things I like about me and things I don't. There are things I can change and things I can’t. I’ve been working on myself for 40 days. Progress is slow and sometimes it feels like I take a step back. For the first time in a long time, I feel good. Not the kratom dragon we were all chasing good, but the real good.”*	*“I’m at my local kratom bar, and I see a new extract on the shelf. I’ve been dabbling in extracts here recently* … *know, I know, they’ll skyrocket my tolerance, they’re addicting, they’re pricey. I use them maybe 3 times a week, no more.”*	*“So, I’ve been experimenting with kratom for years and have only recently learned that freezing a mixture of powdered kratom leaf, water, and citric acid (with a Ph of 4 or below) creates a stronger extraction.”*
*“I’ve quit kratom a few times before. My experiences had ranged from no withdrawal, slightly uncomfortable, to the traditional insomnia/flu like symptoms with mild PAWS (post-acute withdrawal syndrome) after. Normally I could sleep it off and recover with time. Looking back, I usually relapsed either because I made no life alterations, or I experienced some form of PAWS (before I knew what it was) and didn't know how to handle myself.”*	*“Very long-term user (7+ years* … *holy moly). Just quit extracts on a whim about 5 days ago, using powder for now and going to taper from there but holy shit I was not expecting the extract switch to be so horrible.”*	*“I just discovered something interesting about raw honey. When honey is diluted, it begins to produce hydrogen peroxide and the enzymes become activated. Does anybody know if there could be potential to convert mitragynine into 7-hydroxymitragynine if you were to let Kratom sit in a honey water mixture over time?”*
	*“Getting hooked on the extracts was expensive and made my tolerance sky high.”*	
*“I need to thank Kratom for allowing me to hit bottom. Because it drove me to the ground. I’d been flirting with it for a few years, then went full on, eventually becoming a heavy extract user. It stole everything from me.”*	*“Sadly, I didn't turn to powder, capsules, or tea. I turned to a kratom extract shot. (Big mistake). I remember the first experience was getting some after work and then preparing to take it at work first thing in the morning. So, I did this. I had no idea how the euphoria and pain relief was going to be so intense. It felt wonderful at that time. I considered myself lucky to find this herbal supplement that I thought would not be addicting like pain killers. Boy was I absolutely freaking wrong!”*	*“I have been experimenting with many different kratom strains for a long time and their effects with other stimulants and I have found a godly combination* … *I was able to sit down for multiple hours a day and get shit done. Productive as never before. You’re relaxed, chilled but at the same time focused and energetic* … *blend lemons, ginger, honey and optionally some turmeric. Drink it together with good* .. *kratom And finally take some high concentrated ginseng extract. It doesn’t matter if you drink it or chew it as candies although liquid usually have higher concentration. All these different stimulants have amazing reciprocal effects and last for many hours.”*
*“After 3 years of heavy kratom use (3-6 extract shots per day + capsules) and not being able to stay off it for even 1 day at a time, my habit sent me straight to rehab. I’m on day 28 [sobriety] now.”*		
*“I was severely addicted to kratom. I stayed at home all the time, used whenever I wanted for any negative emotion, for any mundane task, for anything/anywhere. I was hopeless. I thought I would never be the same again and never be able to change* … *but I did, and you can too. There’s so much more to this life.”*	*“I put the needle down 4 years ago and was completely sober for a couple years, no kratom, nothing! It has been a year since I’ve been abusing kratom and sneaking around like a fucking junkie. I started with kratom tea then that eventually led me to kratom [liquid extract] shots.”*	
*“[Kratom] initially saved my life from heroin, but after around a year, the negative side effects really became apparent. I isolated. Kratom initially made me very social, but then I felt dumb, uninterested in human contact, became a hermit. And the loss of sex drive? Holy shit! I’m used to that side effect, having been a horrible heroin addict, but somehow, kratom was worse. And the withdrawals! I will admit, I often find some of the quitting kratom posts to be a little alarmist, or deeming a plant evil, simply because one had negative interactions with it. But after years of heavy use, I was amazed at how bad the withdrawals were.”*	*“I’ve been taking kratom* … *capsules and lately liquid extract, for about 5-1/2 to 6 years. Really helped me with getting off the Roxys back then, but I’m now struggling to get away from it. Kratom has cost me untold thousands of dollars, a decent job and is now threatening to rip away my girlfriend of 4 years.”*	
	*“Almost all of that time I was using [kratom] powder only,* … *Until COVID happened and somehow I turned that into an excuse to start buying these little headshop extracts (not the very common ones in the tiny curvy bottle but some other weird brand I can’t find a goddamn thing about online). They were so much cheaper than the other shots that I would buy multiples, and over time that turned into a fucking $40/day habit (sometimes more).”*	

**TABLE 3:** A sample of quotes from Reddit posts (*N* = 370) made between July 2020 to January 2022 detailing kratom use as a drug substitute and consumers’ frustration with the kratom products and the kratom product industry.

Kratom as a drug substitute	Policy talk–frustration with kratom industry and product information
*“Kratom is able to 99% eliminate [of] Fentanyl withdrawal.”*	*“But of course, these alkaloid profiles won't match up name for name with most kratom I could buy right now. If I were to buy these exact names from a random vendor online or in a store then run the same test, it would probably be very different alkaloid profiles. Very frustrating fact of the kratom market.”*
*“I recently tried to [quit opioids] cold turkey 12.5 mcg per hr. fentanyl and was astonished how I am able to function with such little withdrawal. I am only taking 2 grams of Red Bali at a time. This is not my first rodeo. I have been using [intravenous] opiates for 20yrs. I haven't used a needle in 3 years now. It’s a long story but I just wanted to say Kratom is a savior in these moments.”*	*“It may be asking for a lot but my hope for the future is that we can have a real and complete alkaloid profile for every [kratom] batch we buy. Name the batches whatever you want but please just tell me how much of each alkaloid I am getting. That way I can figure out what I like and only buy kratom with those profiles.”*
*“I’m also a ex poly-addict (2+ years clean), and kratom helps my cravings for stimulants.”*	*“If you’re like me -- got sucked into the ‘it’s a harmless herbal supplement/tea’ and were addicted to nothing prior except maybe nicotine/caffeine, and realized you became totally dependent on Kratom and became scared.”*
*“Almost instantly I knew [kratom] was a miracle plant. It took away my craving for alcohol. I’ve had my mishaps here and there since. Kratom always gets me through it. I’m so happy to have found this plant and be a part of this community because the kratom community is so loving and caring. Kratom has been a great tool to help with my depression, anxiety and helping to provide energy on hard days. I couldn't picture a life without it! I know some people get addicted to it and that’s terrible, but I only take 2 grams 3-4 times a day and stick to that. Never going farther. It’s an amazing tool to smooth out the rough edges of life.”*	*“Lately I’ve noticed in most of [podcast name] commercials they advertise SuperSpeciosa kratom- No problem. My issue is how they label it a supplement and never mentions the physically addictive qualities of the drug* ….. *As a kratom user myself, I can tell you first hand that you could easily nod off at the wheel on kratom if you were a novice to the substance. It is an opiate and acts quite similarly to opiates such as Hydrocodone. So basically, they are telling you that you can take this kratom and you won’t feel impaired, even though it can literally make you nod off and pass out from being so high.”*
*“I’m surprised I managed to get to the 2-year [sobriety] mark! I have had cravings and temptations and managed to resist them. Please no replies saying, ‘you’re not really sober’ or ‘you’ve just replaced one thing for another’. I have heard it all before. I have been using kratom for 7 years and it has been nothing but a positive in my life, helping me to finally kick alcohol.”*	*“In my experience this chemical is much more powerful than I gave it credit for and stay here for real information about what it's like I, like many of us, got caught up in the misinformation and downplaying of the negative effects.”*
*“I found Kratom 8 months ago and in that time, I’ve been able to lessen my alcohol use substantially. At first, I was still drinking daily but cut down to less than half the first month. Now I drink zero to one drink on the weekends and none during the week. Sometimes it will be 3 or more weeks before I have a drink. Usually, It’s just socially. Kratom is the only thing that has worked, and I’ve tried it all. Naltrexone gave me horrible side effects, Sobrenix didn't work.”*	*“So instead of a smart person and going to a doctor I remember Kratom is a thing. I looked into it and depending on what you search for and what subreddit you find, you can get heavily conflicting information. So naturally I get the idea that it's basically CBD for opiates and that there wasn't much risk to it, if any, which is quite wrong.”*
*“I can’t even begin to explain how easy it was to transition from an everyday scary ass heroin habit right to brewing teas made from kratom powder. It truly saved my life, no doubt! The first night [of heroin withdrawal] was the worst because I was able to sleep but all night I was sweating in my sleep and waking up drenched and freezing but it got better each and every day. I wanna say that second day was hell when I was waking up because I felt like a dead sleepless zombie and anyone who has gone through the withdrawals on opiates knows the sleep deprivation can be the worst symptom of all* …. *such an evil place in life but with kratom I have found my way back!”*	*“Much of the information and lab testing we see for kratom is coming from vendors and I honestly don't trust most info coming from vendors. Let’s face it, they want to make money, they are in the business of marketing a product to sell it. They don't get together and make any real standardization in regards to the colors and names. The labels are usually some sort of combo of how it looks, the grind, and how it is reported to feel. I’ve had many batches labeled red that are bright green and don't look like they’ve had even the slightest sun exposure and I’ve had greens that look brownish and oxidized. Same with the effects, it is usually all over the place. I’ve tried around a dozen or so of the most popular vendors over the past 6 years and usually get at least 4 different batches to try out. It is a total crap shoot, it is even difficult to get the same kratom from the same vendor months later. I’ve had a few good stints with a batch but eventually the vendor runs out and their new order from Indo isn't the same. They often just slap a name on the different batches and/or make all kinds of blends leading to no real way to know what you are getting.”*
*I was a teenager when the opioid epidemic was starting to really ramp up. Seemed like at the beginning all of my friends parents and grandparents had bottles of OxyContin just sitting in their medicine cabinets. I, like so many others, got hooked. Fast forward 15 years and I’m struggling to deal with life without opiates, I’m having a very hard time quitting. Enter Kratom, a plant related to the coffee tree indigenous to southeast Asia. I used this plant in small doses to help me kick my opiate habit. It worked. I’ve been clean of opiates for over 2 years now, and take kratom occasionally for recreational purposes. I have no dependency on it and only use it a couple times a week max*	*“It’s a safe plant in the coffee family, right? Never trust an article that glorifies a drug, I suppose is an important lesson there.”*
*“I was lost in the void. When I was 22, I decided it couldn’t get any worse* … *By that time I lost many friends to the needle. By that time the damage was done. Hope was fading. I ordered some Kratom not thinking much of it, basically expecting nothing, since weed didn’t even help. Let me tell you this: I was blown away by the effects of this plant* … *I could smile again. I could do what I was supposed to. I had more money in my pocket. I smoked less. Spent more time with my family. Started meditating, facing my anxiety, appreciating every day.”*	*“Being a first-year med student, I was still pretty naive to most of medicine and the gas station down the street from my school used to sell these powdered capsules by the front register. I was talking to the store owner 1 day asking what they were, and he gave me the talk about how they are basically a natural ground up coffee leaf supplement that give people similar energy to regular coffee except without most of the anxiety and jitteriness. This sounded like an absolute no brainer to me, and I felt like I finally found the best coffee substitute in the world that would both give me energy while simultaneously bypassing the side effects of regular caffeine. Of course, stupid me didn't research them at all until I had been taking them for a couple months already, so I had absolutely no knowledge about it being an opioid while I had been taking them every day for months.”*
*“[I’m]Not doing Ice; drinking only beer, kratom, tea and water of course.”*	*“I’m aware that there are plenty of alkaloid[s] in kratom and that th[ese] 2 alkaloids [mitragynine and 7-hydroxymitragynine] are not the only one[s] that play a role in the effects, but it would be a start no? For example, the CBD business did it clean and professional. When you buy a CBD product, it’s written on it: x% of CBD and x% of THC, although it doesn’t mean too much because there are plenty of components in CBD. So why not do the same with kratom? It would give a rough idea of the strength and quality, and maybe we would start to have less bunk. I’m tired of buying always samples of all strains to find out what are the good strains this month. Because let’s be honest, 50% of Kratom around is bunk, and it’s always the magic quest to find the good one a specific time* … *”*
*“I broke my back when I was young and then my neck and shoulder a few years later and did every opiate I could find. Moved to Seattle where there’s more opportunity than ever, slipped a few times* .. *found kratom and it’s saved my life ever since. Just wanna say my relationship with kratom may be unhealthy* … *but it’s helped me live on my own for the first time while holding down 2 jobs and riding BMX every day and doing some super rad shit I’d never be able to endure without some help.”*	
*“Somewhere in there I started drinking a 1-2 whiskies most days a week for about a year before finding kratom. I rarely got drunk but started recognizing it was an everyday thing and needed to be addressed. I don't really remember when I first took kratom, but what I do remember was that when I started kratom, I thought it was a miracle drug and I instantly quit drinking. It was a drug that was a cumulation of the best parts of my some of favorite drugs: weed, coffee, alcohol, and Adderall.”*	*“I stumbled across this when researching 7-Hydroxymitragynine (7-HO). 7-HO is a chemical found in negligible amounts in actual kratom leaf, but it is about 7-10 times more potent than morphine. But since its technically in kratom already, extract companies could conceivably put more of it in the kratom products and say that it’s just a natural part of the kratom, so its ok. It turns out that is just what has been done according to this study. What this means is any kratom products, or leaf, could be “spiked” with more than natural levels of 7-hydroxymitragynine, the potent opioid and analgesic, since there is no oversight* … *I am speculating, but I would guess that these practices or fear of these practices will form the basis for kratom being banned and scheduled.”*
*“I was at the end of my rope trying to deal with neuropathy using methadone. But like so many others, kratom saved the day, and my life.”*	

### 2.3 Network analysis of themes in posts

In service of our aim to examine unique relationships between kratom product types and other experiences, we generated a data matrix of binary variables representing kratom product type and text themes’ occurrence (=1) or not (=0) in each coded text segment for use in a network analysis. Network analysis is a technique to visualize relationships between constructs and within subconstructs; its applications range from describing basic associations to constructing models with interacting paths (Costantini et al., 2015; McNally et al., 2015). The R package qgraph (Epskamp et al., 2012) was used to generate a partial-correlation network of Reddit post themes from a tetrachoric-correlation matrix of binary variables representing each coded theme, thereby visualizing relationships between themes after controlling for the influence of all other themes in the network. The network consists of nodes (one representing each theme) and edges (lines between nodes that represent the partial correlation of those nodes). Edge thickness and color show strength and direction of the partial correlations (green = positive, red = negative); strengths ranged from a minimally relevant effect size (_p_r = 0.10) to the maximum observed (_p_r = 0.47). Nodes are arranged by three measures of centrality: closeness (cumulative strength of association with other themes), betweenness (degree to which themes share partial variance), and strength (degree to which themes moderate associations among other themes).

## 3 Results

We identified 1,443 text segments containing codebook themes in 329 (89%) of the 370 Reddit posts examined, which had a median length of 200.0 words (IQR = 91.0 to 406.8 words). Posts left uncoded, while containing at least one kratom-related search term, did not contain text related to any codebook theme. For all codes applied, 1,209 were concordant and 234 discordant between coders, resulting in an 83.8% agreement. Kappa was 0.82, indicating good interrater agreement beyond chance. We identified descriptions of kratom product types in 134 (36.2%) posts. Leaf products were the most commonly described in these product-type posts (*n* = 63, 17.0%), closely followed by extracts (*n* = 56, 15.1%), and brewed tea (*n* = 33, 8.9%). In terms of reddit communities that these posts originated from, 177 (47.8%) were from kratom subreddits dedicated to general discussion, 96 (25.9%) were from kratom subreddits dedicated to discussing negative kratom experiences, and 197 (53.2%) were from communities dedicated to topics other than kratom.

### 3.1 Prominence of coded themes

Of the 1,443 kratom-related text segments most prominent theme, indicated by the most commonly applied code, was kratom “addiction or dependence” (*n* = 353, 24.5%), which comprised two subcodes: “withdrawal” (*n* = 168, 11.6%) and “tolerance” (*n* = 70, 4.9%). In the codebook, we combined “addiction” and “dependence” into a single code of “professed physical dependence/addiction,” *though we hasten to acknowledge that they are separate constructs in clinical nosology* (see footnote 1). We combined the two constructs because most people made interchangeable use of them and their symptoms, so we were unable to extricate the two reliably. Codes were applied for “quitting, tapering, or reducing” kratom use (*n* = 203, 14.1%) as well as “negative or unwanted effects” from kratom (*n* = 127, 8.8%).

Table 2 shows quotes on kratom addiction, physical dependence, quitting, and extract use. Although we had found indices of perceived addiction and/or physical dependence in prior analyses, they were more prominent here, and the tenor of the discussion was more distressed: “*I was severely addicted to kratom. I stayed at home all the time, used whenever I wanted for any negative emotion, for any mundane task, for anything/anywhere. I was hopeless.”* Kratom-related problems could be characterized as severe for a minority, impairing valued daily roles and obligations:

“*After 3 years of heavy kratom use (3-6 extract shots per day + capsules) and not being able to stay off it for even one day at a time, my habit sent me straight to rehab. I'm on day 28 [*sobriety*] now.”*

Descriptions of kratom-extract use mentioned extracts’ greater potency and greater potential for physical dependence than leaf products:

“*Sadly, I didn't turn to powder, capsules, or tea. I turned to a kratom extract shot. (Big mistake). I remember the first experience was getting some after work and then preparing to take it at work first thing in the morning. So, I did this. I had no idea how the euphoria and pain relief was going to be so intense. It felt wonderful at that time. I considered myself lucky to find this herbal supplement that I thought would not be addicting like pain killers. Boy was I absolutely freaking wrong!”*

*“Almost all of that time I was using [*kratom*] powder only,* … *Until COVID happened and somehow I turned that into an excuse to start buying these little headshop extracts* … *They were so much cheaper than the other shots that I would buy multiples, and over time that turned into a fucking $40/day habit (sometimes more).”*

*“I had been taking Kratom daily for a few years and never had an issue. However, about a year ago I walked into my local smoke shop and finally decided to splurge a bit and bought the two [*extract shots*] for twenty. It all went downhill after that.”*

Another theme we called “kratom science at home” (*n* = 211, 14.6%), comprising three components: “free-form scientific regurgitation” (*n* = 98, 6.8%), characterized by earnest attempts to share scientific knowledge (e.g., links to papers; scientific findings) or to discuss scientific aspects of kratom, often through interpretation of publicly available information; kratom “potentiation and optimization” (*n* = 71, 4.9%), which described methods for altering kratom for greater perceived intensity or duration of effects; and “alkaloid-extraction methods” (*n* = 46, 3.2%), which described processes for isolating kratom alkaloids from plant material. Table 2 provides quotes describing potentiation/optimization, for example:

“*It has been 20 days since I created a mixture of kratom red vein leaf powder, white vinegar, and isopropyl alcohol; shaking daily as the mixtures separate upon resting. The white vinegar takes the mitragynine + 7-hydroxymitragynine, then upon shaking, the isopropyl alcohol takes mitragynine + 7-hydroxymitragynine from the vinegar.”* Also: “*I've been experimenting with kratom for years and have only recently learned that freezing a mixture of powdered kratom leaf, water, and citric acid (with a Ph of 4 or below) creates a stronger extraction.”*

The phenomenon is not new, but the specificity of methods discussed was considerable. Ingredients mentioned for potentiation/optimization included vinegar, lemon juice, grapefruit juice, turmeric, pepper, caffeine, honey, naphtha, and Gotu-kola extract, along with other vitamins, supplements, or spices.

Posts discussing motivations for using kratom sometimes mentioned “self-treatment,” particularly for symptoms of addiction to other drugs (*n* = 99, 6.9%). This included kratom’s use as a “long-term drug replacement/substitute” (*n* = 53, 3.7%) or “short-term drug replacement/substitute” (*n* = 33, 2.3%). Accounts reflected not only self-treatment of opioid-related problems, but patterns of polydrug use that included alcohol, opioids, psychostimulants, and supplements. Quotes illustrating this appear in Table 3, such as:

“*I’m also a ex poly-addict (2+ years clean), and kratom helps my cravings for stimulants”* and “*Almost instantly I knew [*kratom*] was a miracle plant. It took away my craving for alcohol. I’ve had my mishaps here and there since. Kratom always gets me through it.”*

We also coded use of kratom to “self-treat pain” (*n* = 75, 5.2%) or “self-treat psychiatric symptoms” such as anxiety and depression (*n* = 56, 3.9%).

Nearly as common was description of use for enhancement of experience or activity (*n* = 112, 7.8%). The term *enhancement* is variously used in drug-research literature to refer to mood improvement or better physical or cognitive performance. Consistent with those distinctions, we found that use for “recreation” (*n* = 48, 3.3%) was dissociable from use to achieve effects that were “nootropic” (i.e., enhancing memory or cognition; *n* = 46, 3.2%) or “ergogenic” (*n* = 32, 2.2%).

“Policy talk” on kratom (*n* = 72, 5.0%) included discussion about kratom’s regulatory or clinical status internationally (e.g., World Health Organization) and within the US at the federal and state level (e.g., legislation to ban kratom; speculation about the Food and Drug Administration). “Policy talk” also included advocacy (e.g., petitions). “Industry talk” (*n* = 59, 4.1%) was salient in descriptions of perceived inadequacies of kratom vendors or the broader industry:

“*Lately I've noticed in most of [*podcast name*] commercials they advertise [*vendor brand name*] kratom- No problem. My issue is how they label it a supplement and never mentions the physically addictive qualities.” Also: “In my experience this chemical is much more powerful than I gave it credit for.”*

*“So instead of [*being*] a smart person and going to a doctor, I remember Kratom is a thing. I looked into it and depending on what you search for and what subreddit you find, you can get heavily conflicting information. So naturally I get the idea that it’s basically CBD for opiates and that there wasn’t much risk to it, if any, which is quite wrong.”*

These posts (more examples in Table 3) expressed frustrations regarding inconsistency in kratom products’ quality, effects, and labeling. Some voiced grievances about the oversimplification of kratom in advertising and how some advocates portrayed kratom as risk-free. A few posts also discussed ignorance about kratom’s pharmacology among employees at shops that sell kratom.

### 3.2 Results from network analyses

**FIGURE 1:**
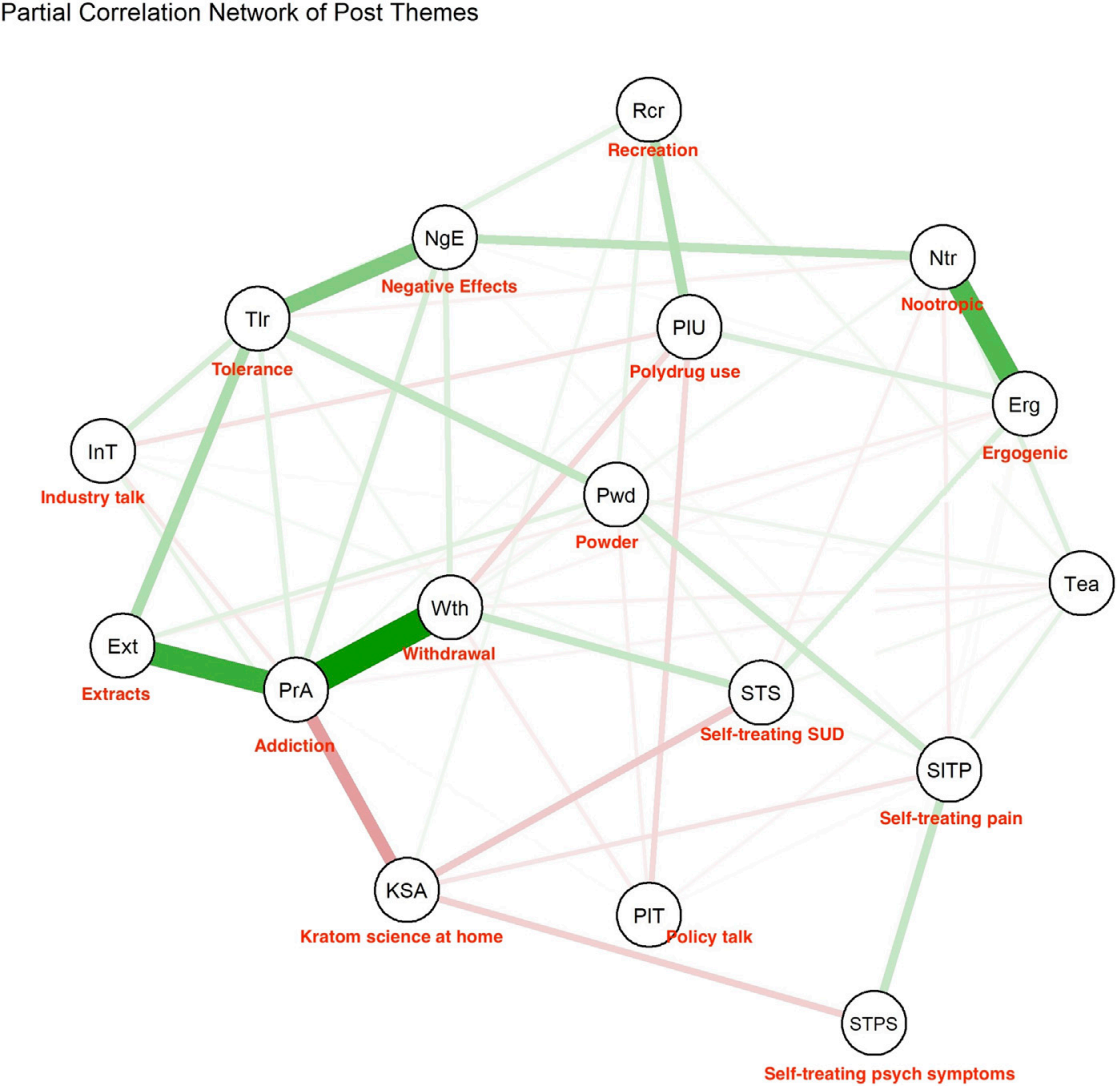
Partial correlation network of post themes. Edge (line) thickness and color show partial correlation strength (range = .10 – .47) and direction (green = positive, red = negative). Nodes are arranged by centrality measures.

Figure 1 shows the partial-correlation network of themes in Reddit posts. The strongest cluster of relationships among codes centered on professed physical dependence/addiction, which displayed a strong positive relationship with descriptions of kratom withdrawal (_p_r = 0.47) but only a small positive relationship with descriptions of kratom tolerance (_p_r = 0.15). Tolerance was most strongly related to descriptions of negative or unwanted effects (_p_r = 0.29), independent of “professed physical dependence/addiction,” and because there was substantial distance between tolerance and professed physical dependence/addiction, people in our sample often discussed tolerance in contexts *other* than those they conceptualized as addiction. In other words, discussion of withdrawal occurred mostly within the context of professed dependence/addiction, whereas discussion of tolerance more often occurred with discussion of other negative or unwanted effects.

Use of kratom extracts was positively associated with professed physical dependence/addiction (_p_r = 0.40) and tolerance (_p_r = 0.22); its location between professed physical dependence/addiction and tolerance in the network indicates that extracts are associated with tolerance independent of professed physical dependence/addiction. Given that “kratom science at home” was negatively correlated with professed physical dependence/addiction (_p_r = −0.24), the positive relationship between extracts and professed physical dependence/addiction was likely driven by use of commercial extracts, an interpretation that seems supported by extract-related quotes (Table 2). Compared with extracts, powder had a weaker positive relationship with tolerance (_p_r = 0.14), and displayed no partial association with kratom withdrawal or professed physical dependence/addiction. Kratom tea was not strongly related to other codes, only displaying a small association with ergogenic effects (_p_r = 0.10). The negative relationship between professed physical dependence/addiction and “kratom science at home” indicates how discussions surrounding kratom optimization rarely accompanied descriptions of physical dependence/addiction.

Kratom withdrawal was weakly but positively related to self-treating symptoms of drug addiction (_p_r = 0.13), including using kratom as a drug substitute. This relationship may reflect the fact that people who reported initiating kratom use to self-treat addiction symptoms often experienced kratom withdrawal symptoms when they tried to discontinue use.

Critically, we could not include “quitting, tapering, or reducing” kratom in the network analysis due to multicollinearity: every “quitting kratom” code co-occurred with a code for “professed addiction,” “tolerance,” or “withdrawal,” which caused network-analysis estimation issues. We return to this in the discussion.

Within the performance-enhancement motivations, kratom use for “nootropic” or “ergogenic” enhancement were distinct from recreation (_p_r = 0.03 and _p_r = −0.03) and highly correlated (_p_r = 0.36). These codes were on opposite sides of the network from professed addiction and displayed strong relationships with each other only, indicating that enhancement discussions were unique from other themes.

## 4 Discussion

We examined Reddit posts made between July 2020 to January 2022, with the initial aim of corroborating and complementing survey-based observations of changes in US kratom products, and examining relationships between product types and consumers’ experiences. The themes we identified speak now to a shift in consumer awareness of risk for a vocal minority of current and former consumers.

### 4.1 Perceived herbal “medicine” or recreational supplement?

We extended prior findings that kratom use can be primarily motivated by “self-treatment” for physical, psychiatric, and addiction-related symptoms (Grundmann, 2017; Swogger and Walsh, 2018; Garcia-Romeu et al., 2020). A departure from prior findings was greater prominence of recreational use, which had not been well characterized (Smith and Lawson, 2017; Coe et al., 2019; Smith et al., 2022c). Although we found instances of recreational use—for relaxation, euphoria, or an intoxicating “high” *without* a secondary self-treatment motivation—we also found recreational use characterized by a desire to enhance other experiences or activities (e.g., exercise, work), without explicitly “self-treating” an underlying issue *and* without the intention of achieving intoxication or a “high.” Kratom use for instrumental enhancement and recreation does not appear to be unique to this sample of Western social media users, as these motivations have been more recently documented in Southeast Asian populations (Singh et al., 2019; 2020). We have also found enhancement motivations in our momentary sampling of regular kratom consumers: many instances of use were driven by proximal desire to “feel good” and increase energy, productivity, focus, and alertness, but not to feel “high” (Smith et al., 2024a). Indeed, intoxication would have likely been viewed as a barrier to performance-enhancing or productivity-enhancing effects. Although we do not know all circumstances of use described in these posts, our EMA investigation found that most kratom use occurred in the earlier part of consumers’ days, without weekend binge use or late-night use. The line between “self-treatment” and use to improve performance, productivity, or quality of life was not always clear, and motivations were not mutually exclusive (e.g., a person may seek energy enhancement and also want to reduce anxiety).

Reports of successful instrumental use were not absent. In many posts, energy- or performance-enhancing use motivations and purely recreational use appear most meaningful when juxtaposed with stated success in achieving intended effects with seemingly little impairment in psychosocial functioning. For those using without the added intention of self-treating medical conditions or addiction, kratom appeared to function as either a vehicle for enhancement and energy or, conversely, as a vehicle for relaxation, but was largely described as part of a normative lifestyle free from kratom-related problems. Our takeaway from these posts is that use motivations, along with “set and setting,” are critical to understanding kratom’s subjective effects, perceived utility, and influence on everyday functioning (Smith et al., 2022e). Despite variability among people, products, and use motivations, effect descriptions consistently indicated that kratom produced (and was sought after for) stimulatory *and* analgesic effects. As kratom products are increasingly broadly marketed, it is likely that motivations will continue diversifying and that consumers will select products they believe will produce desired effects (energy, analgesia, or a combination). Purchasing decisions will remain largely informed by vendor descriptions of effects (i.e., more *versus* less stimulating) and consumer perceptions of kratom veins or branded products amidst market variability and inadequate labeling information (Hill et al., 2023).

### 4.2 Taking pride in part-time chemistry and the kratom community

Descriptions of published scientific findings, along with detailed methods for at-home extractions of kratom alkaloids, were found at a surprising rate, given that surveys have yet to capture these reports. One novel phenomenon was the posting of at-home methods for isolating mitragynine (MG) or 7-hydroxymitraginine (7-HMG, also an active metabolite of MG), two of four kratom alkaloids known to act at mu opioid receptors (Sharma et al., 2019; Berthold et al., 2021; Kamble et al., 2021). Reddit seems to attract people who are able and willing to write meticulous posts describing complex topics or methods, and these kratom-related posts were no exception. These finely described extraction methods are characteristic of some subreddit posts documenting “do-it-yourself” chemistry related to other drugs, such as subversion of abuse-deterrent formulations of opioid analgesics (Sarker et al., 2020; Balsamo et al., 2021). Isolation of kratom alkaloids was usually accompanied with an aim of achieving greater potency for recreational use. Some users posted pictures of their final products, often achieved after trial and error, with evident pride in their craft. Tips were provided to others seeking to perform at-home extractions, and those posting about extraction methods were eager to answer questions.

The practical implications of a possible trend toward do-it-yourself alkaloid extraction are not yet clear. Isolated MG and 7-HMG should not be conflated with whole-leaf kratom. Some kratom leaves have >40 bioactive alkaloids, a diversity likely contributing to kratom’s wide-ranging effects (Hiranita et al., 2022). The potential of each of these compounds to produce euphoria or addiction remains highly speculative, particularly when extrapolating from preclinical work, though some findings suggest that such potential be lower (or negligible) for MG compared to 7-HMG or other alkaloids, such as speciociliatine (Hemby et al., 2019; Kamble et al., 2022).

The theme of “potentiation and optimization” overlapped some with at-home extractions, though the former were often distinguished by people sharing tips or recipes for enhancing or prolonging effects using available ingredients, not isolating alkaloids. Posts detailing extraction methods or describing techniques for potentiating or optimizing kratom’s effects share the commonality that the people posting appeared to sincerely want to distribute knowledge. Cautions were provided, along with acknowledgments that no two kratom batches are alike, nor are any two people. Unlike vendors, possibly motivated by financial incentives, those who posted tips for augmenting kratom effects appeared motivated by a sense of exploration and a desire to help others in what they viewed as a kratom-using community.

### 4.3 Quitting “this insidious weed”

This same sense of community was found among those who had once regularly used kratom but who had quit or reduced use. Nearly all posts that described quitting, or a desire to quit, also involved descriptions of withdrawal, professed addiction, or use that they believed had become problematic. Tolerance was discussed somewhat separately from those topics, a finding that is intriguingly similar to a recent demonstration (in young-adult alcohol drinkers) that tolerance is a separate construct from all other criteria of an SUD (Watts et al., 2021). Problems associated with kratom use were often described as gradual in onset. Onset of unwanted physical dependence was generally associated with extract products (*versus* extracts isolated at home), as evidenced in the network-analysis findings, though it should be noted that extracts were mentioned in only 15.0% of posts and leaf-based products and brewed tea, mentioned in 17.0% and 8.9%. We take these associations as a strong signal that requires systematic investigation. Many described first using kratom powder or tea, often for therapeutic effects, and then trying extracts; few posts described initiating use with extracts.

Discussion around regular extract product use and its problematic outcomes was more frequent than in prior examinations of Reddit posts (Smith et al., 2021a). Extracts were typically reported as more “potent,” not in a strict pharmacological sense (which would refer to compounds with differing effective concentrations at receptors), but in reference to their producing stronger effects at smaller volumes of ingestion. Although some descriptions of extract effects made them sound similar to traditional opioids, other descriptions were mixed. This may partly reflect the inherent effects of mu opioid agonism, which can be subjectively stimulating as well as sedating (Paakkari et al., 1990); it may also reflect the complexities of kratom, including a variety of actions of kratom alkaloids at adrenergic, adenosinergic, and other receptors (Matsumoto et al., 1996; Smith K. E. et al., 2023). We also cannot rule out adulteration or that some kratom products may have contained other ingredients (e.g., kava, caffeine, cannabidiol). Likewise, we cannot know the extent to which extract products were co-used with other psychoactive substances, supplements, prescription drugs, or over-the-counter medications (Smith et al., 2024a).

It may be worth clarifying that we (and most other authors) use the term *extract* as shorthand for *concentrated extract*. Brewing whole-leaf kratom into tea with water is essentially a method of extraction, but it is one of the least efficient methods, decocting a solution with a relatively low concentration of alkaloids (Grundmann et al., 2024). Consuming whole-leaf powders by the spoonful, or as prepared capsules containing leaf material, also leads to extraction of alkaloids, but only by the consumer’s stomach acid—a slow and, again, relatively inefficient process. The products we call *extracts* are made with methods that are highly efficient in “pulling out” higher concentrations of leaf materials into small volumes of liquid, and tend to be readily absorbable and bioavailable (Avery et al., 2019). These properties may profoundly shorten the time of onset of effects, and raise their peak intensity. We know of no studies in humans characterizing the pharmacokinetics and pharmacodynamics of kratom extract products.

Here, nearly all consumers who had used extracts *and* expressed a desire to quit, or who had quit, described indicators of perceived physical dependence or addiction. The desire to quit was also associated with other problems in physical and psychosocial functioning presumed to be kratom-related (e.g., hair loss, low testosterone, isolation, secrecy about use, anxiety, cost), irrespective of product type (whole-leaf and extracts). Although whole-leaf kratom forms were also discussed unfavorably among those who had quit kratom, they were not discussed with the same fervor. Tea was seemingly viewed as the most benign kratom preparation, and in keeping with traditional use preparations (Jayadeva et al., 2017).

### 4.4 A tale of two plants, two products, and the limitations of reddit

Indicators of kratom physical dependence/addiction were contained nearly exclusively on the subreddit “r/quittingkratom,” the virtual space dedicated to the topic. The general “r/kratom” was not a dedicated space for discussing quit attempts or successes and was more diverse in content and tenor. It is difficult to overstate the difference between the content found on the two subreddits. If readers unfamiliar with kratom were to read posts on “r/kratom,” they would have difficulty characterizing kratom in a single sentence, given the nuance and variety of posts reflecting variability in product formulations, use patterns, and perceived benefits and risks. This variability was reflected in agnosticism, ambivalence, curiosity, and more favorable or tempered attitudes about kratom in “r/kratom.” Most likely, readers would come away with the impression that “kratom,” generically speaking, is a mild psychostimulant, like coffee, or mild partial opioid agonist, or a combination of the two, with more benefit than risk. If the same readers were to read “r/quittingkratom” instead, they would likely come away with the impression that kratom is a full opioid agonist with more risk than benefit; use of “kratom” would appear to be an entirely different, even extreme, experience. We should not discount the experiences of people self-selecting to engage with either forum, but we should understand that the generation of discussion posts is motivated as a contribution to the particular topic (subreddit) at hand. For r/quittingkratom, the topic was necessarily focused on a very narrow set of experiences, many of which were complicated. Indeed, many who reported quitting kratom had tremendous self-insight and described a complex set of factors complicating their lives, kratom being one of many, and not the first. Animus towards kratom or the kratom industry was least pronounced among those who posted about their problems with kratom while *simultaneously* clarifying that many of their self-assessed problems pre-dated kratom use initiation.

In our own survey work, we now make specific efforts to reach respondents who have stopped using kratom, including people who post to the “r/quittingkratom” subreddit or are active in the anti-kratom organizations. We encourage designers of kratom-related surveys to include these potential respondents, along with current, presumably satisfied, consumers, in their study recruiting.

### 4.5 A Meta perspective and other limitations

In the years since our Reddit data collection, “anti-kratom” voices, though possibly fewer in number than “pro-kratom” voices, have established a robust online presence, critical not only of kratom vendors, lobbyists, and advocates, but also independent scientists investigating kratom. As we analyzed only 370 posts, we are reluctant to generalize too strongly about perceptions of kratom, favorable or unfavorable. Importantly, our use of Reddit data confers an inherent demographic bias. People posting to Reddit are most likely to be between 18 and 29 years old (44%) and male (27% vs 17% female; Pew Research Center, 2024). Although those demographics align well with at least some aspects of the demographics of kratom use (Rogers et al., 2021), those choosing to post to Reddit are, by definition, highly invested in a topic, and typically have strong opinions on the topic. Likewise posts, however descriptive, are only snapshots in time of a person’s thoughts and feelings. This has the potential to create the impression that there are only two extremes of kratom-use experiences when, in reality, there is likely a great silent majority of kratom consumers who neither post to Reddit nor self-select to survey participation. We believe studies should seek to sample from both extremes, as well as those in the expanse between, and who may be harder to enroll into scientific studies.

### 4.6 Conclusion: the reckoning and continued uncertainty to come

A takeaway from this study also underscores a limitation, which is the significant variability of what is called “kratom.” Our findings suggest that conclusions about real-world kratom use via self-report are best drawn by specifying the kratom *product* type and dose. When we were first analyzing these data, we were inclined to distinguish concentrated extracts from whole-leaf products along a fairly straightforward continuum of higher to lower risk of adverse effects. This still holds as a broad generalization, but we also think more nuance is needed. In the Reddit posts we analyzed, we usually could *not* know what types of “extracts” were used (e.g., isolated MG, full- or broad-spectrum), their concentrations, and the serving sizes recommended or used. This means that even within the “kratom extract” category of products, further differentiation is required to make sense of findings. Indeed, even “brewed tea” is an aqueous extraction, though we do not believe posters describing “extracts” would consider brewed kratom tea as an extract. Likewise, kratom leaves are pharmacologically complex and diverse even prior to processing. Their commodification and eventual product diversification only add greater uncertainty regarding what is meant when studying “kratom.” With the increasing proliferation of kratom products, the era of assessing self-reported effects from “kratom” generically should conclude. The era of assessing effects from specific *kratom products* must begin in earnest. Critically, US kratom use should not be presumed to resemble the traditional kratom preparation and use methods found in Southeast Asia. Self-reported kratom use and use assessed in case reports should be *further* differentiated by type (whole-plant product *versus* extract), preparation (e.g., brewed tea *versus* encapsulated powder), dose, and co-ingestants (Smith et al., 2022e).

The variability of products, and the inconsistent effects produced, were discussed by consumers posting to Reddit as an industry failure. Some kratom vendors were viewed with disappointment and frustration regarding product consistency, quality, and information provided, sentiments we have found among some in other qualitative work (Smith K. E. et al., 2023). This can be contrasted with views of liquid extracts: they were not explicitly described as inconsistent in their effects, but they were more likely to be described as producing physical dependence. Although most consumers do not appear to want kratom criminalized, and express concern over prohibitions, many want industry improvements and some regulation to ensure product quality, safety, and consistency. Some state-level legislation has been or is actively being introduced to provide some regulation, but this does not change the fact that kratom and other products currently persist in a gray market.

We are abstaining from policy recommendations, but we do recommend greater transparency from kratom vendors. It may be difficult to standardize all product types, but vendors should acknowledge limitations and unknowns directly, list major alkaloids on the package, include recommended serving sizes based on safety studies, and provide warnings when appropriate. Absent regulatory oversight, it is critical that kratom is not advertised as merely another “herbal supplement” without no potential for physical dependence. Ideally, decision-making by individual consumers would be done with medical consultation, particularly when use is for self-treatment of chronic conditions (Swogger and Walsh, 2018). Ultimately, our findings suggest that kratom products, during this 2020-2022 period, were deemed to be helpful for some and a hindrance to others, but that, irrespective of use motivations or outcome, consumers were frustrated and placed at risk by a regulatory wilderness that they were, and are, largely navigating on their own.

## Data Availability

The data analyzed in this study is subject to the following licenses/restrictions: Post data were downloaded from the social-media platform Reddit, when Reddit’s data were publicly accessible via requests to their application programming interface (API). On April 18, 2023, Reddit began to restrict access to their API as part of an initial public offering, and we have not obtained permission to publish full post data. Data sets containing generated text theme by reddit post are available upon request. Requests to access these datasets should be directed to JR, jmrogers@health.ucsd.edu.

## Footnotes

1 Per the DSM-5, substance-use disorders (SUDs) are diagnosed for specific classes of substances, but there is a “not otherwise specified” category for substances not listed, such as kratom; the name of the substance is to be specified in the diagnosis. We therefore refer to kratom-use disorder (KUD) as a diagnostic entity. We also use KUD synonymously with “kratom addiction.” For any SUD, the DSM-5 states that symptoms of physical dependence (tolerance, withdrawal, or use to avoid withdrawal) can contribute to the SUD diagnosis but are not necessary for it and are not always sufficient for it.
